# The aging-related risk signature in colorectal cancer

**DOI:** 10.18632/aging.202589

**Published:** 2021-02-26

**Authors:** Taohua Yue, Shanwen Chen, Jing Zhu, Shihao Guo, Zhihao Huang, Pengyuan Wang, Shuai Zuo, Yucun Liu

**Affiliations:** 1 Division of General Surgery, Peking University First Hospital, Peking University, Beijing 100034, People’s Republic of China

**Keywords:** colorectal cancer, TCGA, GEO, aging, risk signature

## Abstract

Background: Colorectal cancer (CRC) is the third most common cancer worldwide. The opening of the TCGA and GEO databases has promoted the progress of CRC prognostic assessment, while the aging-related risk signature has never been mentioned.

Methods: R software packages, GSEA software, Venn diagram, Metascape, STRING, Cytoscape, cBioPortal, TIMER and GeneMANIA website were used in this study.

Results: Aging-related gene sets, GO_AGING, GO_CELL_AGING and GO_CELLULAR_SENESCENCE, were activated significantly in CRC tissues. We constructed an aging-related risk signature using LASSO COX regression in training group TCGA and validated in testing group GSE39582. The risk score was significantly associated with the overall survival of CRC patients, whose stability was clarified by stratified survival analysis and accuracy was demonstrated using the ROC curve. The risk score was significantly increased in the advanced stage, T3-4, N1-3 and M1 and positively correlated with the richness of immune cell infiltration in the tumor microenvironment. We further investigated the molecular characteristics of 15 hub genes at the DNA and protein levels and performed GSEA between high- and low-risk groups.

Conclusions: The aging-related signature is a reliable prognostic analysis model and can predict the severity and immune cell infiltration of CRC patients.

## INTRODUCTION

According to the 2020 Colorectal Cancer Statistics, in the United States, colorectal cancer (CRC) is the second most common cause of cancer death [1]. The early symptoms of CRC are not obvious. As cancer grows, bowel habits change, including blood in the stool, diarrhea, alternating diarrhea and constipation, and local abdominal pain [2].

The latest report points out that the age of onset of CRC is getting younger, with the median age dropping from 72 years in 2001-2002 to 66 years in 2015-2016. However, new cases are still predominantly middle-aged and elderly, accounting for 88% of new diagnostic CRC patients in the United States in 2020. After age stratification, for people over 55 years old, the incidence rate increases by about 30% for each increase of 5 years old [1].

Population aging is a typical feature of many developed countries. The association between aging and cancer is becoming more and more apparent [3]. Aging is an important biological process and a general feature of biological organisms. The major manifestations of aging are the gradual loss of function or degeneration at the molecular, cellular, tissue and body levels [4]. Aging is an essential risk factor for cancer, as well. One of the characteristics of aging is hyperplasia, the most serious of which are cancers. Besides, cancer, like other aging-related diseases, mostly begins at the midpoint of life [5]. Identifying the key features of senescence and induction of senescence in cancer cells has been considered in anti-cancer research [6]. Impaired antitumor immunity is a typical example of immune aging [7]. Immune-related risk signature [8], hypoxia-related signature [9], autophagy score signature [10] and somatic mutation signatures [11] have been constructed to predict the overall survival (OS) of CRC patients. However, the aging-related risk signature to predict the survival of CRC patients has never been built.

In this study, based on the Gene Set Enrichment Analysis (GSEA), we found that the aging-related gene sets, GO_AGING, GO_CELL_AGING and GO_CELLULAR_SENESCENCE, were activated significantly in CRC tissues. Next, we extracted 49 aging-related gene sets at the Molecular Signatures Database v7.1, from which we collected 1693 aging-related genes shared by the training group TCGA and testing group GSE39582. In the TCGA data set, we mined 258 differentially expressed aging-related genes and 26 prognostic aging-related genes. Based on the LASSO regression analysis, the 15-gene-aging-related risk signature was constructed, significantly associated with the OS of CRC patients in both the training and testing groups. Survival analysis stratified by age, gender, American Joint Committee on Cancer (AJCC) stage, tumor size *in situ* (T), lymph node metastasis (N) and distant metastasis (M), clarified the stability and independence of aging-related risk signature. Univariate and multivariate COX regression analysis further confirmed that our risk signature was an independent prognostic factor. At the same time, the Receiver Operating Characteristic (ROC) curve demonstrated the accuracy of this risk signature. The aging-related risk score was significantly increased in the advanced stage, T, N and M and positively correlated with the degree of 5 types of immune cell infiltration. The 15 aging-related genes included in the signature were named hub genes. We further investigated the mutation and copy number alteration (CNA) of these hub genes at the DNA level. At the GeneMANIA website, we mined functionally similar genes of hub genes and constructed a protein-protein interaction network. Functional enrichment showed that these hub genes and functionally similar genes were mainly involved in platelet alpha granule and regulation of endopeptidase activity, consistent with results in plasma proteomic signature of age in healthy humans. In terms of hallmark gene sets and KEGG gene sets, we performed the gene set enrichment analysis (GSEA) between high- and low-risk groups to understand our risk signature’s molecular mechanisms and pathways.

In conclusion, the aging-related risk signature can predict the OS, severity and immune cell infiltration of CRC patients. 15 hub genes are expected to become novel therapeutic targets in the future.

## RESULTS

### Compared with normal mucosa, the aging process of cells was activated significantly in colorectal cancer

**Figure 1:**
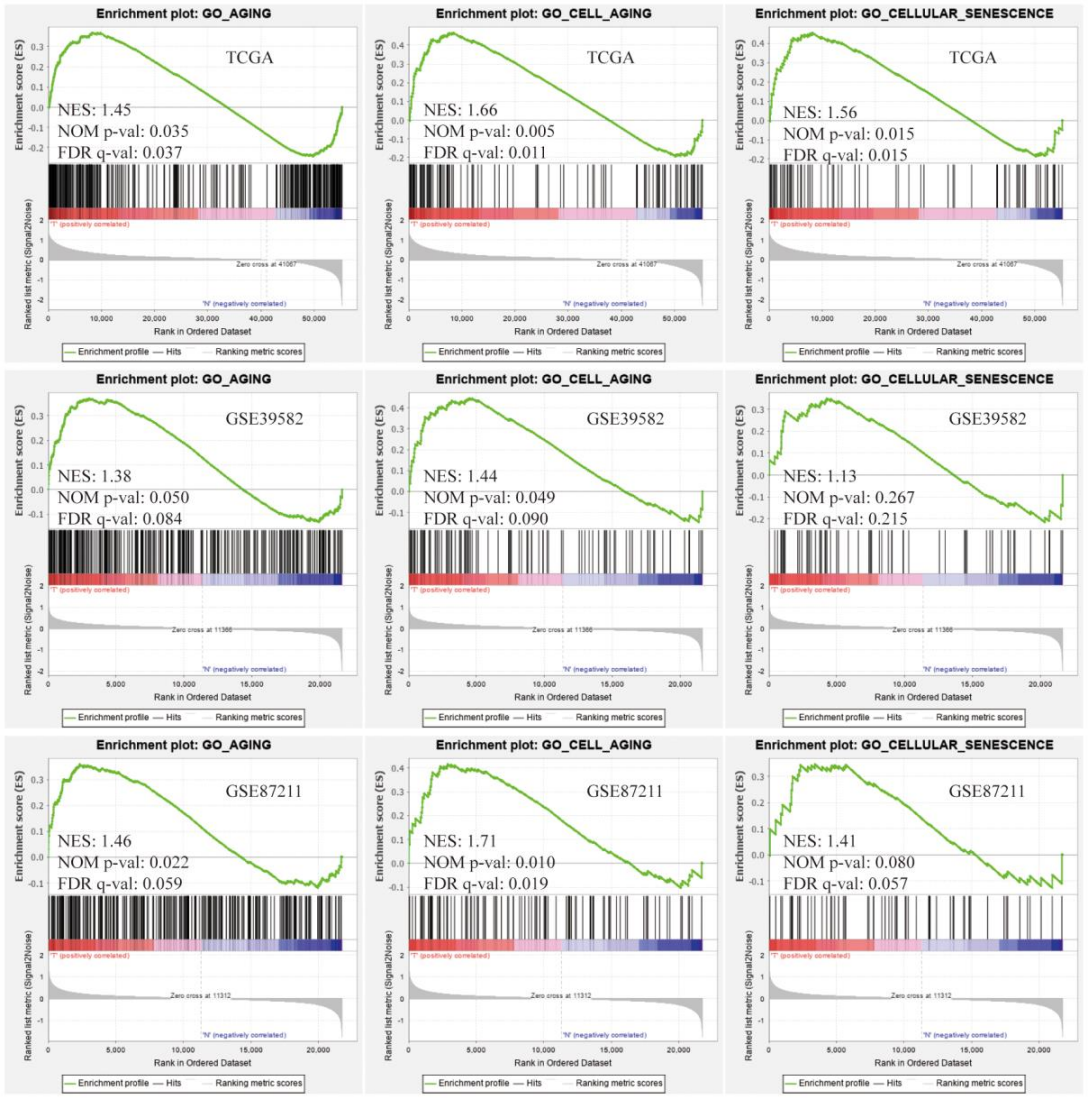
**Gene Set Enrichment Analysis (GSEA).** Three aging-related gene sets were significantly activated in colorectal cancer (CRC) tissues compared with normal tissues. The significance criteria were nominal P-value < 5% and FDR q-value<25%.

In vertebrates, aging leads to degenerative and proliferative pathological changes, the most fatal of which was cancer. Based on 3 independent data sets, TCGA, GSE39582, GSE87211 (Table 1), we conducted the Gene Set Enrichment Analysis (GSEA) on the gene sets of "GO_AGING, GO_CELL_AGING and GO_CELLULAR_SENESCENCE" and found that the aging-related gene sets were significantly activated in colorectal cancer (CRC) tissues compared with normal tissues (Figure 1). The above results showed that the aging process was involved in the development of CRC and suggested that aging-related signature might predict the survival of CRC patients.

**Table 1:** Number of samples and genes in colorectal cancer data set.

**Data sets**	**Numbers of genes**	**Normal tissues**	**Tumor tissues**
TCGA	56753	44	568
GSE39582	21655	19	566
GSE87211	21754	160	203

### Identification of aging-related genes

The inherently complex biological changes of aging, as well as inflammation, immune aging, oxidative stress, and age-related chronic diseases, have crucial impacts on the development and deterioration of malignant tumors. At the Molecular Signatures Database v7.1, we searched 49 gene sets related to aging (Table 2). 1837 aging-related genes were finally included in this study.

**Table 2:** Aging-related gene sets included in this study.

**Name (Number of genes)**	
DEMAGALHAES_AGING_DN (16)	DEMAGALHAES_AGING_UP (54)
GO_AGING (323)	GO_CELL_AGING (117)
KIM_HYPOXIA (19)	GO_CELLULAR_SENESCENCE (78)
KYNG_DNA_DAMAGE_DN (184)	KYNG_DNA_DAMAGE_UP (209)
KYNG_NORMAL_AGING_DN (18)	KYNG_NORMAL_AGING_UP (15)
LY_AGING_MIDDLE_DN (16)	LY_AGING_MIDDLE_UP (14)
LY_AGING_OLD_DN (56)	LY_AGING_OLD_UP (7)
LY_AGING_PREMATURE_DN (30)	GO_MUSCLE_ATROPHY (12)
RODWELL_AGING_KIDNEY_DN (146)	RODWELL_AGING_KIDNEY_UP (500)
KYNG_WERNER_SYNDROM_DN (18)	KYNG_WERNER_SYNDROM_UP (15)
KYNG_WERNER_SYNDROM_AND_NORMAL_AGING_DN (194)	
KYNG_WERNER_SYNDROM_AND_NORMAL_AGING_UP (80)	
RODWELL_AGING_KIDNEY_NO_BLOOD_DN (147)	
RODWELL_AGING_KIDNEY_NO_BLOOD_UP (223)	
WEIGEL_OXIDATIVE_STRESS_BY_HNE_AND_H2O2 (36)	
WEIGEL_OXIDATIVE_STRESS_BY_HNE_AND_TBH (59)	
WEIGEL_OXIDATIVE_STRESS_BY_TBH_AND_H2O2 (35)	
WEIGEL_OXIDATIVE_STRESS_RESPONSE (33)	
GO_MULTICELLULAR_ORGANISM_AGING (35)	
GO_NEGATIVE_REGULATION_OF_CELL_AGING (28)	
GO_POSITIVE_REGULATION_OF_CELL_AGING (23)	
GO_REGULATION_OF_CELL_AGING (60)	
GO_REPLICATIVE_SENESCENCE (14)	
GO_STRIATED_MUSCLE_ATROPHY (9)	
KYNG_DNA_DAMAGE_BY_4NQO (36)	
KYNG_DNA_DAMAGE_BY_4NQO_OR_GAMMA_RADIATION (15)	
KYNG_DNA_DAMAGE_BY_4NQO_OR_UV (60)	
KYNG_DNA_DAMAGE_BY_GAMMA_AND_UV_RADIATION (81)	
KYNG_DNA_DAMAGE_BY_GAMMA_RADIATION (78)	
KYNG_DNA_DAMAGE_BY_UV (56)	
KYNG_ENVIRONMENTAL_STRESS_RESPONSE_DN (17)	
KYNG_ENVIRONMENTAL_STRESS_RESPONSE_NOT_BY_4NQO_IN_OLD (12)	
KYNG_ENVIRONMENTAL_STRESS_RESPONSE_NOT_BY_4NQO_IN_WS (37)	
KYNG_ENVIRONMENTAL_STRESS_RESPONSE_NOT_BY_GAMMA_IN_OLD (29)	
KYNG_ENVIRONMENTAL_STRESS_RESPONSE_NOT_BY_GAMMA_IN_WS (31)	
KYNG_ENVIRONMENTAL_STRESS_RESPONSE_NOT_BY_UV_IN_OLD (23)	
KYNG_ENVIRONMENTAL_STRESS_RESPONSE_NOT_BY_UV_IN_WS (12)	
KYNG_ENVIRONMENTAL_STRESS_RESPONSE_UP (53)	
MA_PITUITARY_FETAL_VS_ADULT_UP (0)	

### Identification of differentially expressed and prognostic aging-related genes in the training group

**Figure 2:**
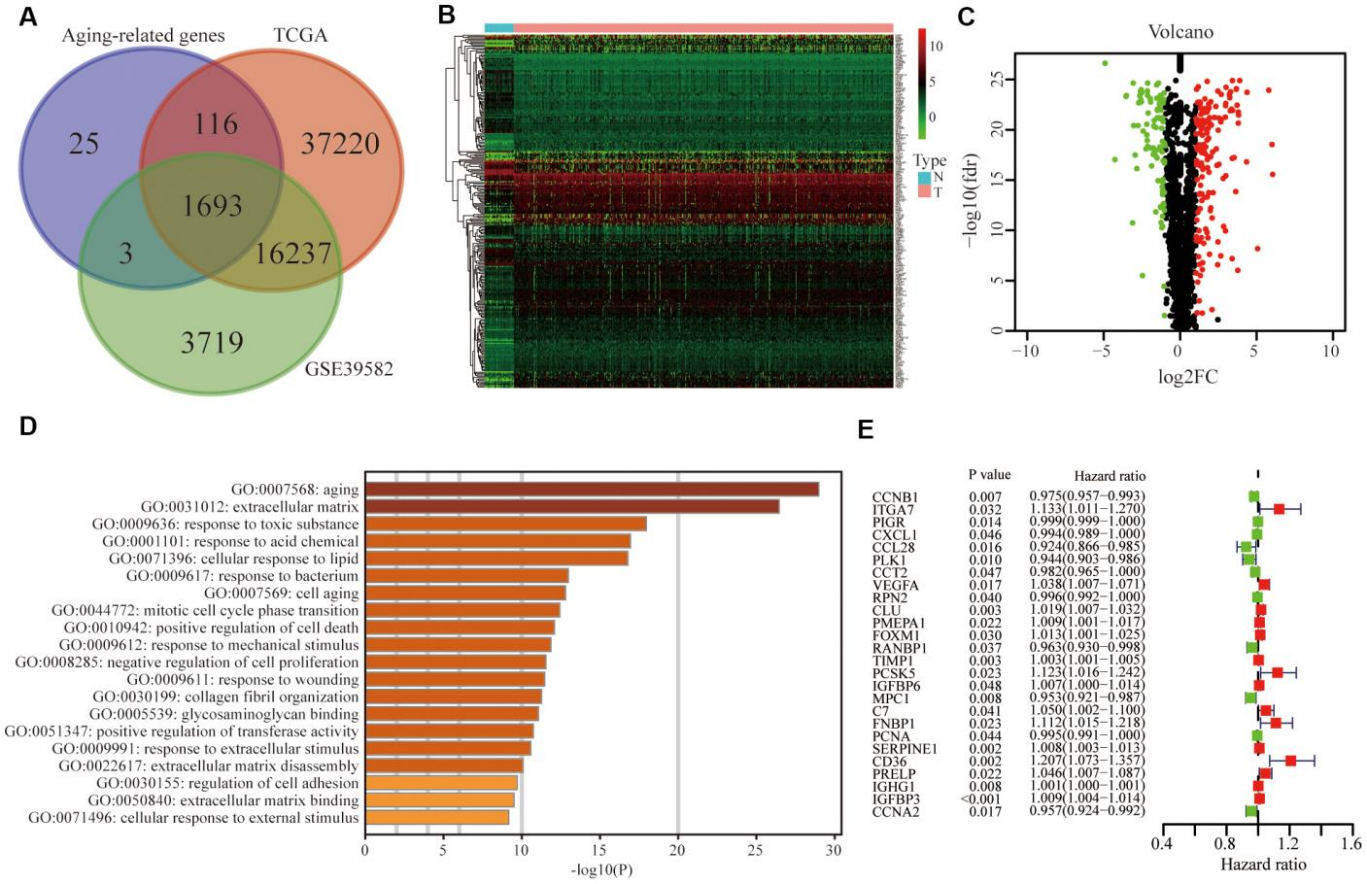
**Identification of differentially expressed and prognostic aging-related genes in CRC.** (**A**) Aging-related genes shared by the training group TCGA and testing group GSE39582. (**B**) Differentially expressed aging-related genes in the TCGA was displayed in the heatmap and (**C**) the volcano map. (**D**) Gene ontology (GO) analysis of these genes. (**E**) Forest plot of prognostic aging-related genes in the training group.

Based on the online Venn diagrams tool, we screened 1693 common aging-related genes in the TCGA and GSE39582 databases (Figure 2A). The former was the training group while the latter was the testing group. Among 1693 genes, we further extracted 258 differentially expressed genes in the TCGA and illustrated them in the heatmap (Figure 2B) and volcano map (Figure 2C), including 164 up-regulated and 94 down-regulated. As expected, the Gene Ontology (GO) analysis revealed that 258 differentially expressed genes were mainly involved in aging (Figure 2D). To predict the prognosis of CRC patients, among these 258 genes, 26 survival-related genes were screened and displayed in the forest plot (P <0.05), including 15 risky genes (HR > 1) and 11 protective genes (HR < 1) (Figure 2E).

### Protein-protein interaction and enrichment analysis among 258 aging-related genes

**Figure 3:**
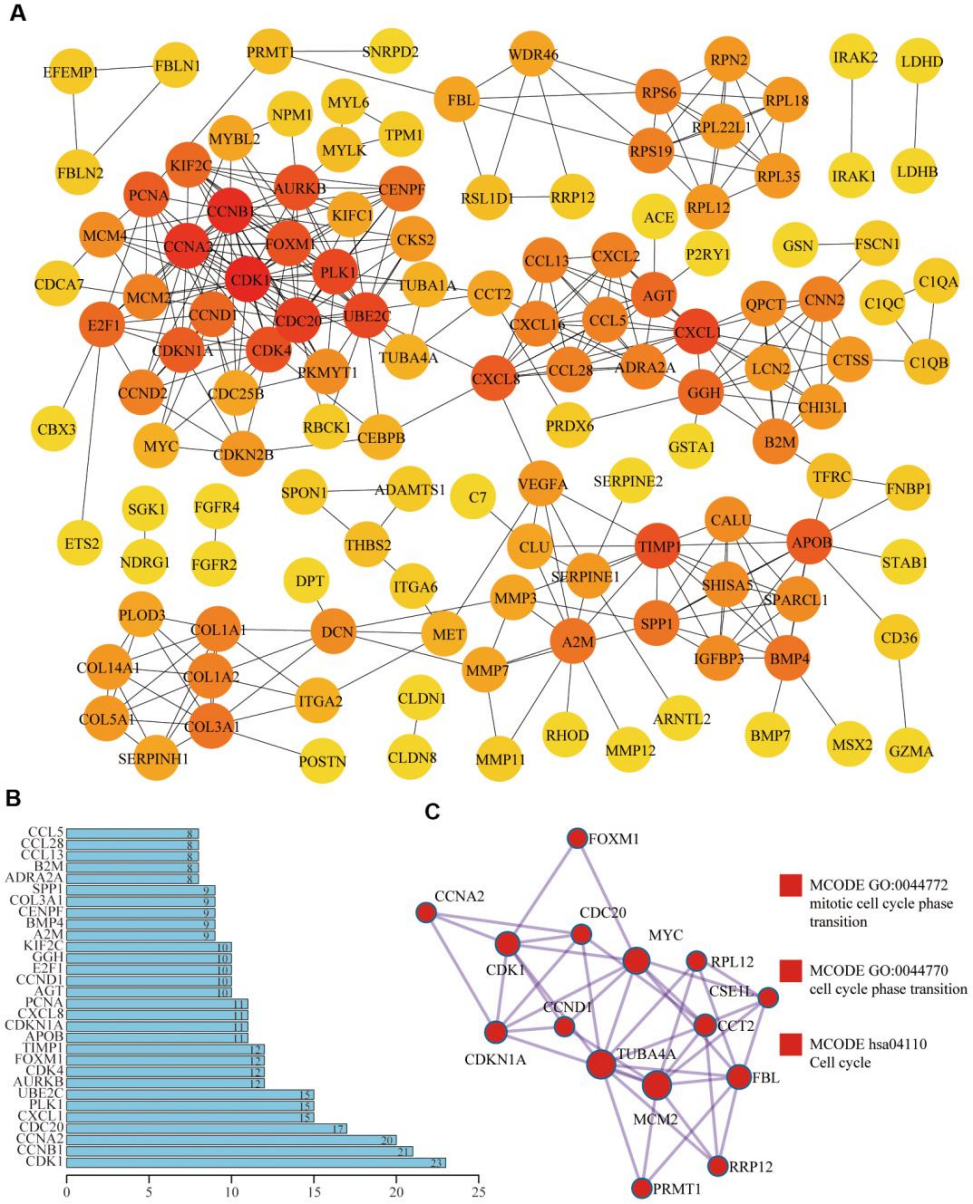
**Protein-protein interaction (PPI) of differentially expressed aging-related genes.** (**A**) In the PPI network, the darker the color was, the greater the number of neighboring nodes was. (**B**) Top 30 genes with the most neighboring nodes. (**C**) The first MCODE component identified in this gene list and pathway and process enrichment analysis of this MCODE component was significantly related to the cell cycle.

Based on the STRING database and Cytoscape plugin cytohubba, we constructed a protein-protein interaction (PPI) network (Figure 3A). The darker the color was, the greater the number of neighboring nodes was. The top 30 genes with the most neighboring nodes were shown in Figure 3B. Next, we performed PPI enrichment analysis at the Metascape and screened the first MCODE components, which were mainly enriched in the cell cycle (Figure 3C). On this website, the legend of the same MCODE component was shown in the same color.

### Development of aging-related risk signature

**Figure 4:**
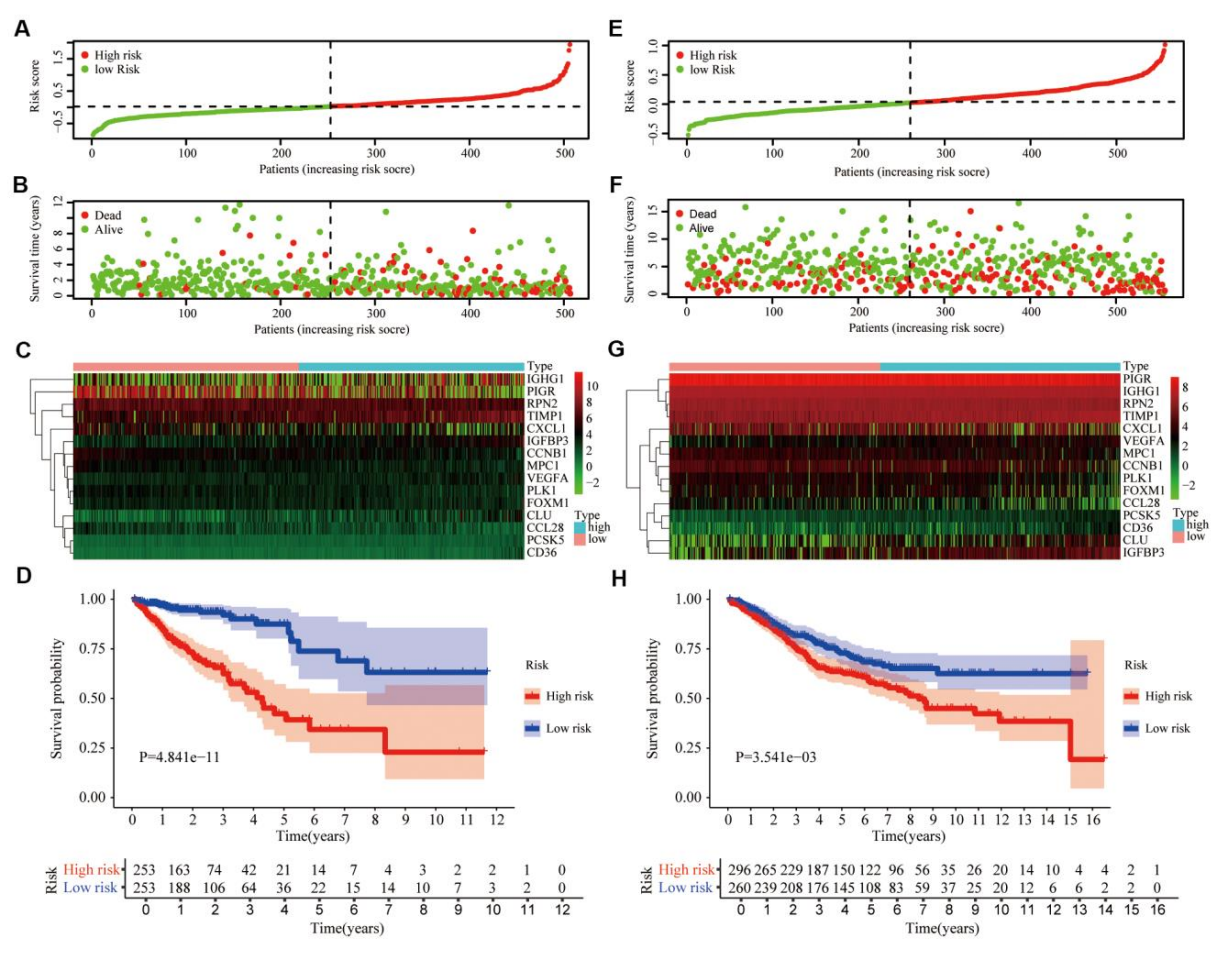
**Prognostic signature based on 15 hub genes.** (**A**) Distribution of groups based on the aging-related risk score. (**B**) The scatter plot demonstrated the differences in the survival status of CRC patients between high- and low-risk groups. (**C**) Heatmap showed differential expression of included 15 hub genes in both groups. (**D**) The overall survival (OS) of the high-risk group was significantly shorter than that of the low-risk group. (**E**–**H**) The second verification was performed in the testing group GSE39582.

Based on TCGA data set, univariate COX and LASSO analysis, we established an aging-related risk signature of CRC patients. The explicit formula of aging-related risk signature was as follow: CCNB1 expression *(-0.00482) + PIGR expression *(-0.000151) + CXCL1 expression *(-0.000198) + CCL28 expression *(-0.00104) + PLK1 expression *(-0.0130) + VEGFA expression *0.0201 + RPN2 expression *(-0.00195) + CLU expression *0.00171 + FOXM1 expression *0.0117 + TIMP1 expression *0.00144 + PCSK5 expression *0.0167 + MPC1 expression *(-0.00826) +CD36 expression *0.0405 + IGHG1 expression *1.33e-05 + IGFBP3 expression *0.00373. The 15 genes included in the model were named hub genes. In terms of the training group TCGA, according to the median of the risk score, we equally divided CRC patients into 2 groups, low- and high-risk groups (Figure 4A). The number of deaths of CRC patients in the high-risk group was significantly higher than that in the low-risk group (Figure 4B). Heatmap showed the differential expression of hub genes between high- and low-risk groups (Figure 4C). We also confirmed that the overall survival (OS) of the low-risk group was significantly longer than that of the high-risk group (P<0.05) (Figure 4D).

To demonstrate the universality of the risk signature, based on the median of risk score in the training group TCGA, we divided CRC patients of the testing group GSE39582 into low- and high-risk groups (Figure 4E). The number of deaths of CRC patients in the high-risk group was slightly higher than that in the low-risk group (Figure 4F), and hub genes were also differentially expressed between these 2 groups (Figure 4G). Next, we draw the same conclusion that the aging-related risk signature was significantly associated with the OS of CRC patients in the testing group (P<0.05) (Figure 4H).

### Validation of the aging-related risk signature

**Figure 5:**
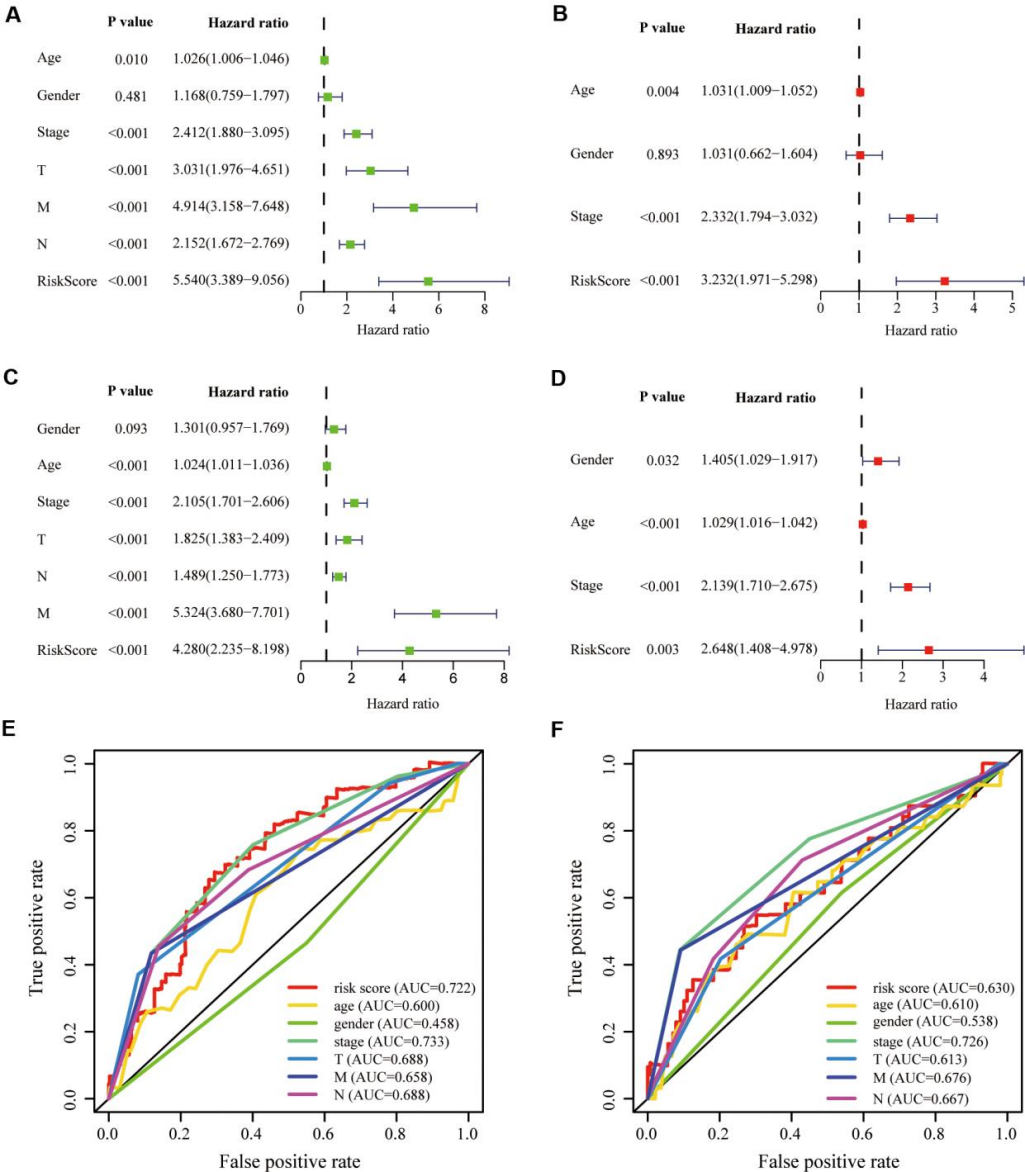
**Validation of prognostic signature.** (**A**, **B**) Univariate and multivariate COX regression analysis in the training group. (**C**, **D**) Univariate and multivariate COX regression analysis in the testing group. The receiver operating characteristic (ROC) curve and the areas under the curve verified the accuracy of prognostic signature in the (**E**) training and (**F**) testing groups.

To investigate whether our aging-related risk signature was a clinically independent prognostic factor, we performed univariate independent prognostic analysis and found that our signature was an independent prognostic factor, like stage, T, N and M, in the training group (Figure 5A). Next, we conducted multivariate independent prognostic analysis and revealed that in the training group, stage and risk score were independent prognostic factors (Figure 5B). Second verification was performed in the testing group GSE39582 and we draw the same conclusions (Figure 5C, 5D). The above results suggested that the aging-related risk signature could be used to predict the survival of CRC patients. To exclude the effect of multicollinearity between stage and T, N, M, T, N and M were not included in the multivariate independent prognostic analysis.

To evaluate our signature’s prediction accuracy, we plotted the Receiver Operating Characteristic (ROC) curve and calculated the areas under the curves (AUC). Finally, we concluded that the risk score performed well in the training and testing groups, compared with T, N and M (Figure 5E, 5F).

### Stratified survival assays

**Figure 6:**
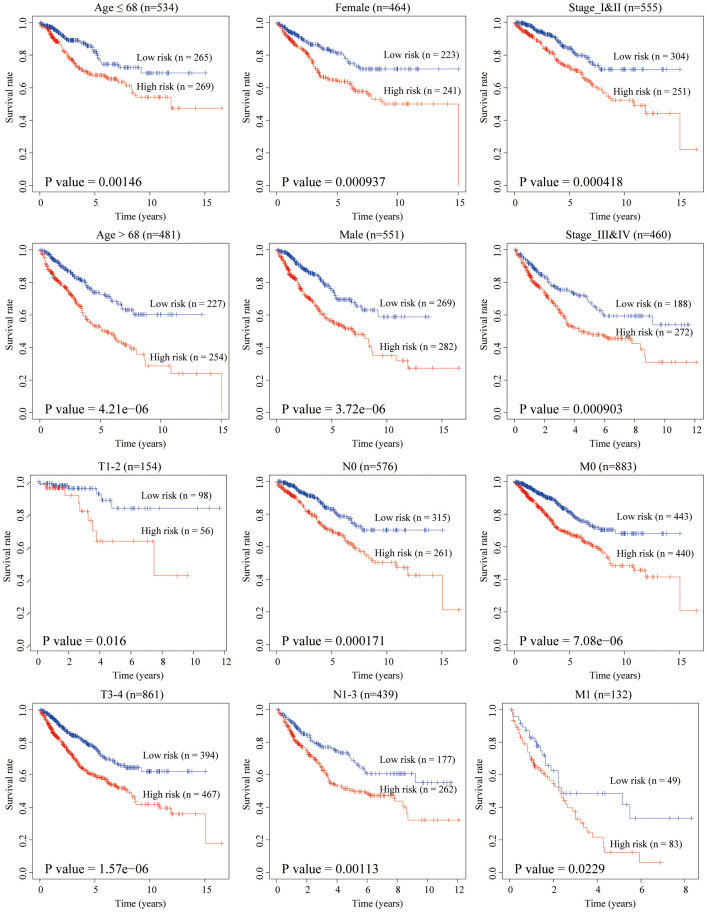
**Stratified survival analysis adjusted to age, gender, stage, T, N and M.** All CRC patients in the training and testing groups were summarized in the stratified survival analysis. 68 years old was the median age of 1,015 CRC patients.

The clinical characteristics of the training and testing group were illustrated in Table 3. To verify our model’s stability and independence in different clinical subgroups, we performed the stratified survival analysis of 1015 CRC patients adjusted to age, gender, stage, T, N and M using the aging-related risk score. All CRC patients in the training and testing group were summarized in the stratified survival analysis. Finally, we concluded that our signature had an excellent prognosis stability. Results of stratified survival analysis were displayed in Figure 6.

**Table 3:** Clinical characteristics of the training group TCGA and the testing group GSE39582.

**Clinical characteristics**		**Number and percentage of CRC patients**	
		**TCGA (%)**	**GSE39582 (%)**
**Total**		490	525
**Age (years)**	≤68	264 (53.9%)	270 (51.4%)
	>68	226 (46.1%)	255 (48.6%)
**Gender**	Male	266 (54.3%)	285 (54.3%)
	Female	224 (45.7%)	240 (45.7%)
**Stage-AJCC**	I&II	277 (56.5%)	278 (53.0%)
	III&IV	213 (43.5%)	247 (47.0%)
**T**	T1-2	100 (20.4%)	54 (10.3%)
	T3-4	390 (79.6%)	471 (89.7%)
**N**	N0	286 (58.4%)	290 (55.2%)
	N1-3	204 (41.6%)	235 (44.8%)
**M**	M0	417 (85.1%)	466 (88.8%)
	M1	73 (14.9%)	59 (11.2%)

*68 years old is the median age of 1,015 CRC patients in our study.

### Pearson correlation analysis between 15 hub genes

To clarify whether there was any epistasis among the 15 hub genes, by summarizing the training set and validation set data, we conducted a Pearson correlation analysis between 15 hub genes, and results showed that all the correlation coefficients were less than 0.6, which showed that the problem of gene epistasis could be ignored. The correlation diagram was shown in Supplementary Figure 1. Red and blue represented the negative correlation and positive correlation, respectively. Both values and dots size represented the Pearson correlation coefficient. * indicated the statistical difference and P-value was set to 0.05.

### Clinical relevance of risk signature

**Figure 7:**
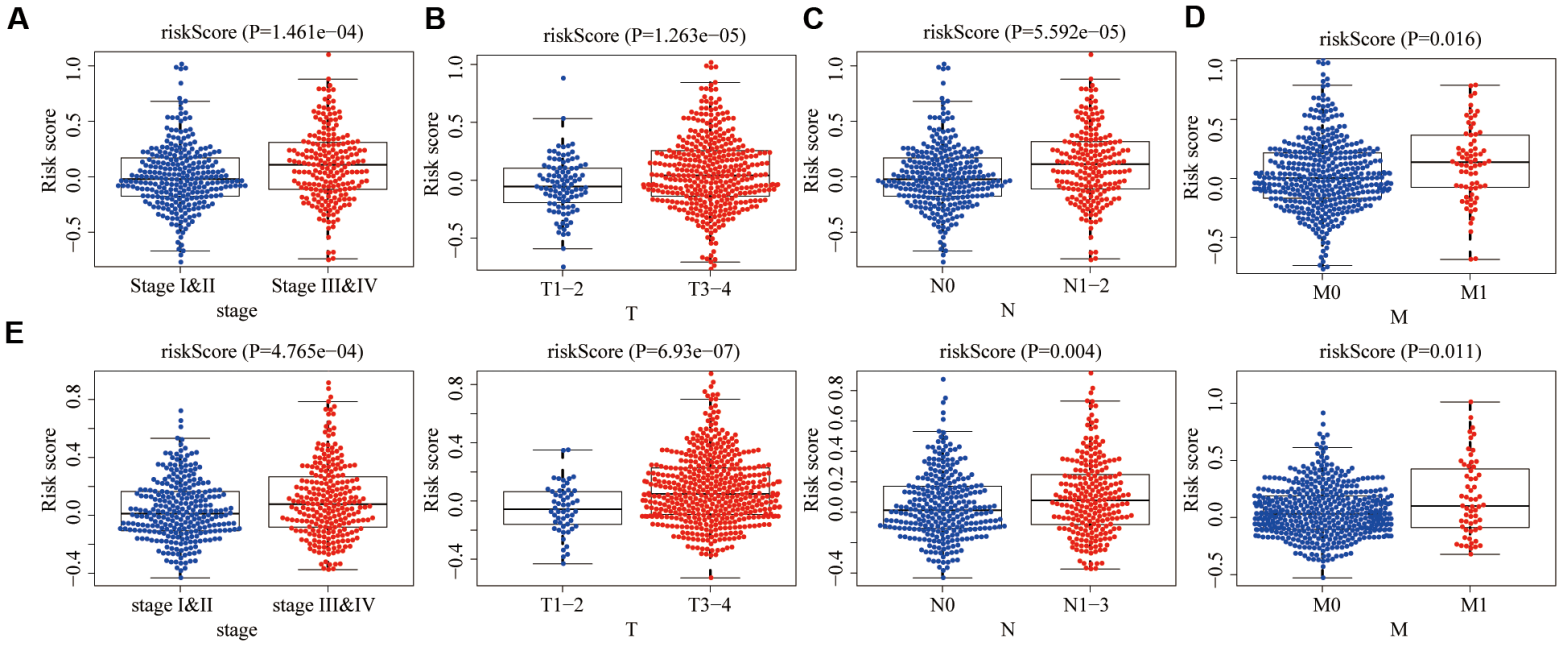
**Relationships between risk score and clinicopathological traits.** The aging risk score of (**A**) stage III & IV, (**B**) T3-4, (**C**) N1-3 and (**D**) M1 were significantly higher than that of stage I&II, T1-2, N0 and M0 in the training group (P < 0.05). (**E**) The same conclusion was obtained in the testing group.

In the training group, we assessed the relevance between the aging-related risk score and clinicopathological traits, including stage, T, N and M. The aging-related risk score was significantly increased in advanced stage cases (Figure 7A), advanced T stage cases (Figure 7B), positive lymph node metastasis cases (Figure 7C) and positive distant metastasis cases (Figure 7D). In the testing group, we got the same conclusion (Figure 7E). These results were consistent with the shorter OS of the high-risk group. Compared with other clinicopathological traits, we could clearly find that the P-values of 'M' were relatively less significant. The above results meant that care should be taken when judging whether CRC patients have distant metastases based on the score.

### Correlations with immune cell infiltration

**Figure 8:**
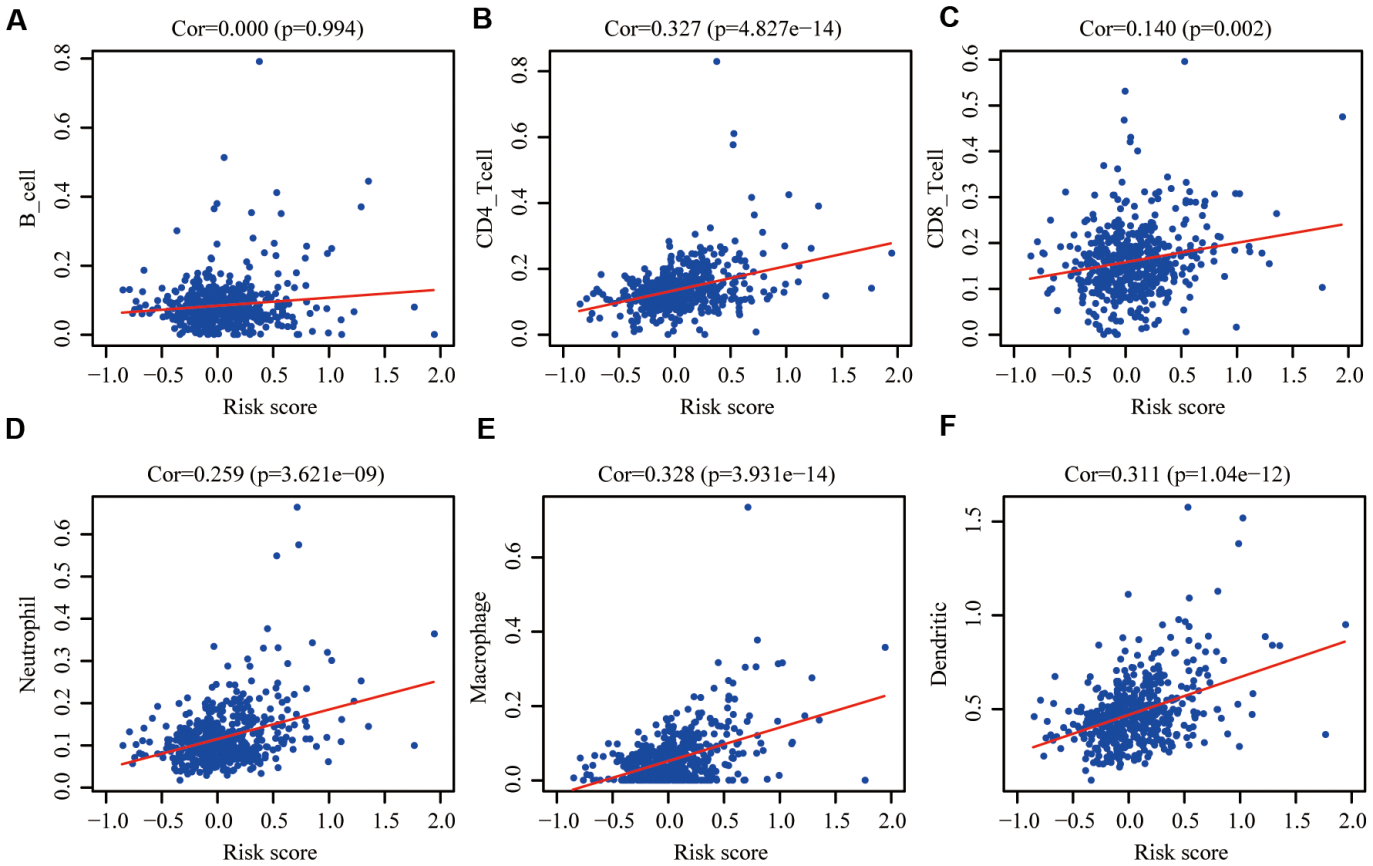
**Pearson correlation analysis between the risk score and infiltration abundances of 6 types of immune cells in the training group.** (**A**) B cells; (**B**) CD4+T cells; (**C**) CD8+T cells; (**D**) neutrophils; (**E**) macrophages and (**F**) dendritic cells. P < 0.05 was considered statistically significant.

One of aging characteristics is immune remodeling, which mainly included T cell immunosenescence and immune dysregulation. In order to determine whether this risk signature accurately reflected the immune status of the CRC tumor microenvironment, we evaluated the relationships between the infiltration of 6 types of immune cells and the aging risk score in the training group. We found that except B cells (Figure 8A), five types of immune cells, CD4^+^T cells (Figure 8B), CD8^+^T cells (Figure 8C), Neutrophils (Figure 8D), macrophages (Figure 8E) and dendritic cells (Figure 8F), were positively correlated with the aging risk score (P<0.05).

### Mutation and copy number alteration (CNA) analysis of 15 hub genes

**Figure 9:**
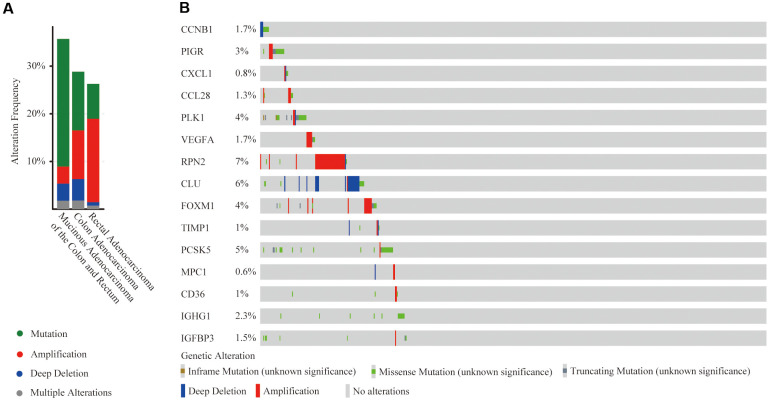
**Mutation and copy number alteration (CNA) analysis of hub genes.** (**A**) Frequency of mutation and CNA in hub genes in 3 types of CRC patients; (**B**) Mutation and CNA of each hub gene.

At the cBioPortal, we found that these 15 hub genes were altered in 152 (29%) of 526 patients/samples (TCGA, PanCancer Atlas), specifically 35.71% of 56 cases with mucinous adenocarcinoma of the colon and rectum, 28.83% of 333 cases with colon adenocarcinoma patients and 26.28% of 137 cases with rectal adenocarcinoma (Figure 9A). The amplification of RPN2 and the deep deletion of CLU were the most frequent CNA among these 15 hub genes (frequency >5%), while there was no amplification or deep deletion changes in IGHG1 (Figure 9B).

### Protein-protein interactions (PPI) of hub genes at the GeneMANIA

**Figure 10:**
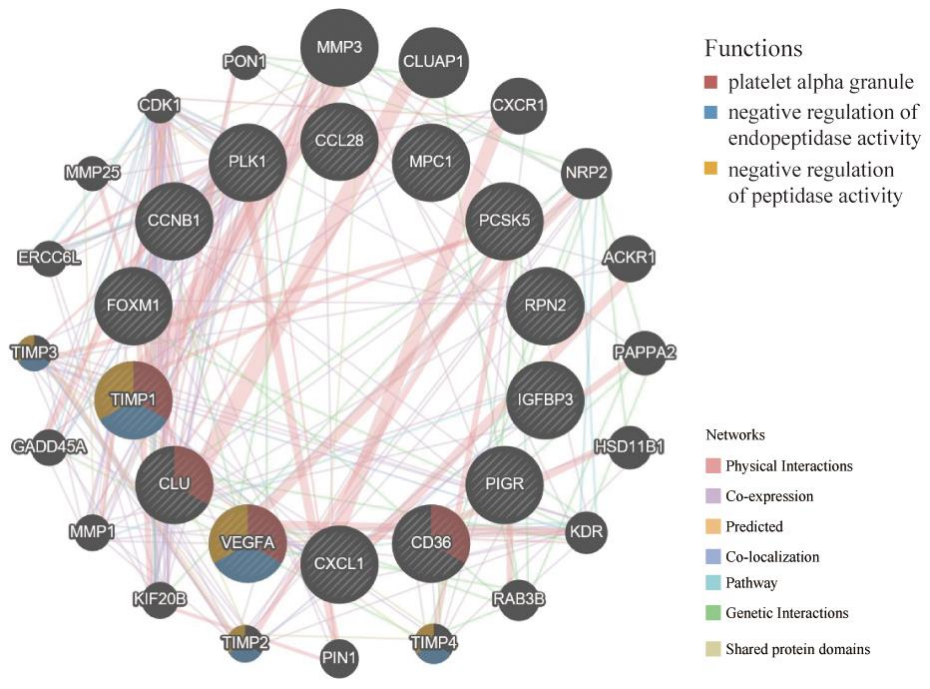
**GeneMANIA website was used to identify functionally similar genes and establish a PPI network.** The 20 functionally similar genes were located in the outer circle, while hub genes were located in the inner circle. The color of nodes was related to the protein function while line color represented the type of protein interaction.

The GeneMANIA is used to predict functionally similar genes of hub genes. We obtained 20 similar genes of hub genes (Figure 10). The hub genes were located in the inner circle, while the predicted genes were in the outer circle. Their functions focused on platelet alpha granule and endopeptidase activity, which coincided with the previous study of functional pathways of age-related proteins [12]. The release of platelet alpha granule increases during blood coagulation, and blood coagulation is the main functional pathway of age-related proteins.

### Molecular characteristics and pathways of the aging-related risk signature

**Figure 11:**
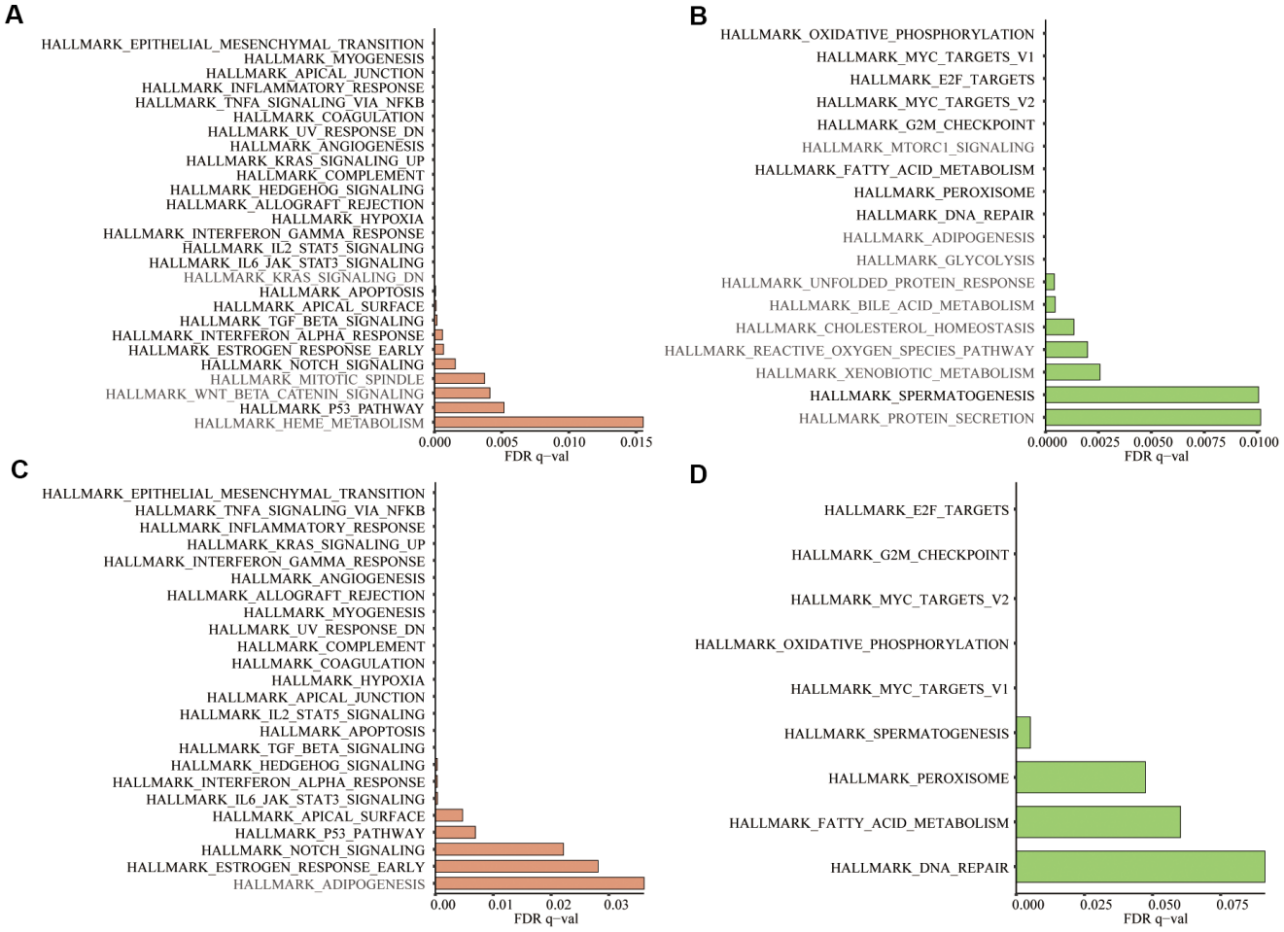
**In the 50 hallmark gene sets, we conducted GSEA between the high- and low-risk groups.** (**A**) Significant enrichment of 27 hallmark gene sets in the high-risk group of training group TCGA; (**B**) Significant enrichment of 18 hallmark gene sets in the low-risk group of the training group; (**C**) Significant enrichment of 24 hallmark gene sets in the high-risk group of testing group GSE39582; (**D**) Significant enrichment of 9 hallmark gene sets in the low-risk group of the testing group. Dark black represented the enrichment results common to both datasets (Nominal P-value < 5% and FDR q-value<25%).

Based on the training and testing data sets, we conducted GSEA on 50 hallmark gene sets and 178 KEGG gene sets between low- and high-risk groups. For the hallmark gene sets, 23/50 gene sets were commonly upregulated in the high-risk group and 9/50 gene sets were commonly upregulated in low-risk groups (Figure 11A–11D). For the KEGG gene sets, KEGG_BASAL_CELL_CARCINOMA were commonly upregulated in high-risk groups, while none gene sets was commonly upregulated in low-risk groups. The filter criteria for enrichment was nominal P-value < 5% and FDR q-value<25%.

## DISCUSSION

Colorectal cancer (CRC) remains the third most commonly diagnosed cancer, ranking second of cancer-related mortality. Due to changes in living habits and population aging, CRC incidence is increasing continuously [13].

As the public databases of the TCGA, GEO and Molecular Signatures Database (MSigDB) are freely available, increasing risk models have been established to prejudge the overall survival (OS) of CRC patients. However, among the various risk signatures, the aging-related risk signature has never been mentioned.

Our study identified 1693 aging-related genes shared in both the training group TCGA and testing group GSE39582 and further constructed a 15-gene risk signature using the LASSO COX regression analysis based on the TCGA data set. The aging-related risk score was significantly associated with the OS of CRC patients and higher in the advanced AJCC stage, T stage, N stage and M stage, whose accuracy for the prediction of the OS was credible based on the ROC curve and the area under the curve (AUC). We also confirmed that this aging-related risk signature was positively correlated with the degree of immune cell infiltration in the CRC tumor microenvironment. All in all, we could predict the survival, severity and immune cell infiltration of CRC patients based on our risk signature. The same conclusions were obtained in the testing group.

Biologically speaking, aging is an inevitable process that occurs spontaneously in organisms over time. It is manifested by the degenerative changes of structure and the decline of function, adaptability and resistance. Physiologically, aging is regarded as the history of individual development from the beginning of the fertilized egg to old age. Pathologically, aging is the results of stress, injury, infection, declining immune response, malnutrition, metabolic disorders and drug abuse. Aging is considered as an independent risk factor for many chronic diseases and most common malignancies, including CRC [6]. The mutation accumulation theory of aging partially explains the relationship between aging and cancer [14]. In cancer research, malignant tumors and aging can be seen as two aspects of the same underlying cellular and molecular process [15]. Aging promotes carcinogenesis, tumor progress and resistance to cancer therapy [16]. Inflammation, one of the seven pillars of aging, increases the risk of cancer and leads to the initial mutation of genes and metastasis of cancer [17]. Immunosuppression promotes age-related impairment of antitumor immunity, which is a typical example of immune aging [7]. Novel markers of aging also have prognostic potentials for cancer [18]. Besides, senescence also has tumor-suppressing functions, owing to the persistence of growth stagnation caused by senescence, which paves the way for cancer treatment [19]. The above facts indicate that there is an urgent need to improve our understanding of the relationship between aging biology and cancer.

Among 15 hub genes, ribophorin-II (RPN2) had a higher amplification frequency. Previous studies have revealed that increased RPN2 is significantly associated with poor histological differentiation, advanced stages and lymph nodes metastasis in patients with CRC [20]. RPN2 promotes CRC cell proliferation by upregulating the glycosylation of EGFR [21]. Moreover, the downregulation of RPN2 can induce apoptosis and inhibit migration and invasion [22]. Among 15 hub genes, the deep deletion of clusterin (CLU) was the most obvious. Previous studies have shown that high CLU mRNA expression levels in CRC patients often represent a poor outcome. Some controversial data have been published and reveals its dual faces as a tumor suppressor or a pro-survival factor in CRC [23]. Besides, Immunoglobulin Heavy Constant Gamma 1 (IGHG1) had no copy number changes in CRC patients. The role of IGHG1 in CRC has never been investigated. In prostate cancer research, inhibition of IGHG1 can suppress cell growth and induce cell cycle arrest and ultimate apoptosis [24].

In the study of plasma proteomic signature of age in healthy humans, functional pathways of age-related proteins mainly included blood coagulation, chemokines and inflammatory pathways, axon guidance, peptidase activity, and apoptosis [12]. At the GeneMANIA, hub genes and predicted 20 similar genes were mainly focused on blood coagulation and endopeptidase activity, which indicated that hub genes were associated with the aging process.

Our research’s advantage is a large number of samples in the TCGA and GEO databases to identify and verify risk signature. The limitation of our study is that only retrospective studies have been performed. Thus, a cohort of CRC patients will be needed to test this signature in the future.

CRC is getting younger and younger, and it is urgent to make accurate survival prediction for diagnosed patients and make better treatment strategies. We first constructed and verified the aging-related risk signature in different data sets. This risk score was significantly increased in patients with advanced AJCC stage, T3-4, positive lymph node metastasis and positive distant metastasis. Compared with other clinical parameters, the P-values of "M" is relatively insignificant. We need to be cautious when determining whether CRC patients have distant metastases based on this score. Moreover, this risk score was significantly and positively correlated with the richness of 5 types of immune cell infiltration in the tumor microenvironment.

Taken together, our prognostic signature can predict the severity of CRC and the level of immune cell infiltration. Aging-related risk signature will be a novel prognostic assessment tool, and the 15 hub genes also need more functional analysis to explore their possible clinical values.

## MATERIALS AND METHODS

### Data collection

In this study, the transcriptome profiling data and corresponding clinical information of colorectal cancer (CRC) were downloaded from The Cancer Genome Atlas (TCGA) (https://portal.gdc.cancer.gov/) and Gene Expression Omnibus (GEO) (https://www.ncbi.nlm.nih.gov/geo/). The samples with follow-up time less than 30 days were excluded to reduce the interference of unrelated factors. 49 aging-related gene sets, 50 hallmark gene sets and 178 KEGG gene sets were downloaded from the Molecular Signatures Database v7.1 (https://www.gsea-msigdb.org/gsea/index.jsp) [25, 26]. The data on the immune cell content of 32 tumors in the TCGA database was downloaded from the Tumor-Infiltrating Immune Cells (TIMER) website (https://cistrome.shinyapps.io/timer/) [27].

### Gene set enrichment analysis (GSEA)

Table 1 showed the number of CRC tumor samples, normal samples and detected genes in 3 data sets, TCGA, GSE39582 [28] and GSE87211 [29]. The GSEA was a computational method that could predict whether the defined set of genes showed statistically significant, concordant differences between 2 biological conditions [30]. To study the role of aging in CRC, GSEA was performed to analyze the enrichment of GO_AGING, GO_CELL_AGING and GO_CELLULAR_SENESCENCE between tumor samples and normal samples via GSEA software version 4.0.1. To investigate the aging-related risk signature’s molecular characteristics and pathways, we conducted GSEA to displayed differences between high- and low-risk groups in 50 hallmark gene sets and 178 KEGG gene sets. Significance criteria were nominal P-value <5% and false positive rate (FDR) <25%. Gene set permutations were performed 1,000 times for each analysis.

### Differential gene analysis

With the help of the Venn diagrams tool (http://bioinformatics.psb.ugent.be/webtools/Venn/) and the R software “limma” package (http://www.bioconductor.org/) [31], 258 differentially expressed aging-related genes were extracted from the TCGA database. P<0.05 and logFC>1 were set as filter criteria. Heatmap and volcano plot were used to visualize differential genes.

### Metascape

The Metascape (http://metascape.org) is a friendly, reliable tool for functional enrichment analysis [32]. The number of min overlaps and min enrichment were 3 and P-value cutoff was 0.05. Next, we performed the protein-protein interaction enrichment analysis on this website, and the Molecular Complex Detection (MCODE) algorithm had been applied to determine densely connected network components [33]. Pathway and process enrichment analysis had also been used to each MCODE component independently.

### Protein-protein interaction (PPI)

The STRING website (https://string-db.org/) integrated and constructed the PPI using computational predictions, which visualized the intrinsic links among 258 differentially expressed aging-related genes [34]. Cytoscape's plugin CytoHubba was used to discover key nodes in the PPI network [35].

### Development of the prognostic signature based on the LASSO COX regression

Univariate COX and LASSO-penalized COX regression were used to construct optimal prognostic risk signatures for CRC samples in the training group. The COX regression model with the LASSO penalty successfully achieved compression and selected 15 aging-related genes simultaneously [36]. The risk score formula was as follows: $\text{risk}\;\text{score}=\sum_{\text{i}=1}^{\text{n}}\text{expiβi}$ where exp represented the gene expression value while β represented the LASSO coefficient.

### Validation of the predictive value of the aging-related signature in both the training and testing groups

In both the training and testing groups, based on the median of risk score, CRC patients were divided into high- and low-risk groups. Scatter plots was used to display the survival status of CRC patients of the low-and the high-risk group. Utilizing the “pheatmap” package, a heatmap was constructed to show the differential expression of hub genes between the low-and the high-risk groups. The R packages “survival” and “survminer” were used to explore the optimal cut-off of risk score and drawn the Kaplan-Meier survival curve. Age stratification was based on the median age of 1015 CRC patients. The two-sided log-rank P < 0.05 was considered statistically significant for survival analysis.

### Pearson correlation analysis

With the help of “corrplot” packages, we had drawn the correlation map, which reported Pearson correlation values between 15 hub genes.

### Evaluating signature performance in training and testing groups

Aging-related risk signature and clinical parameters, including age, gender, stage, T, N and M, were considered as covariates. We performed univariate independent prognostic analysis. In order to avoid multicollinearity between stage and T, N, M, the multivariate independent prognostic analysis only included gender, age, stage and risk score. Results were illustrated in the forest plots. Green and red represented univariate and multivariate independent prognostic analysis, respectively. P-value, hazard ratios (HR) and 95% CI of each variable were also displayed in the forest plots. The “survivalROC” package was applied to confirm the predictive accuracy of the risk signature [37].

### The clinical correlation and the correlation of immune cell infiltration

Differences between risk signature and clinicopathological parameters stage, T, N and M were tested using independent t-tests [38]. The Tumor IMmune Estimation Resource (TIMER) database analyzes the richness of immune cell infiltration in the tumor microenvironment [27]. The current version of TIMER incorporates 11510 samples across 32 cancer types of TCGA database [38]. 6 types of immune cells are B cells, CD4 T cells, CD8 T cells, neutrophils, macrophages and dendritic cells. Spearman correlation analysis was performed between aging-related risk score and immune cell infiltration. P-values of less than 0.05 were considered statistically significant.

### Mutation and copy number alteration (CNA) analysis of hub genes

The cBioPortal (https://www.cbioportal.org) is a friendly website exploring, visualizing and analyzing multi-dimensional cancer genomic data [39]. The copy-number alteration (CNA) and mutation of 15 hub genes were identified using segmentation analysis and GISTIC algorithm in the cBioPortal among 526 CRC patients/samples (TCGA, PanCancer Atlas) [40].

### GeneMANIA

The GeneMANIA website (http://genemania.org) is used to predict functionally similar genes of hub genes and construct the PPI network among them [41]. It can also predict the relationships among functionally similar genes and hub genes, including protein-protein, protein-DNA interactions, pathways, physiological and biochemical reactions, co-expression, co-localization [42]. In this study, we explored functionally similar genes of hub genes and performed functional enrichment analysis.

### Statistical analysis

Statistical analysis in this study were performed with R software 3.6.1. P <0.05 and logFC>1 were the filtering criteria for differential genes. LASSO regression analysis was utilized to exclude highly correlated aging-related genes and prevented the signature from overfitting. The Kaplan-Meier survival curves were constructed to analyze survival differences between the low- and high -risk groups. Student's t-test was used to determine the relationships between the risk score and clinical parameters. Based on univariate and multivariate COX proportional hazard models, we calculated the hazard ratios of prognostic factors and screened independent prognostic factors. The ROC curve was used to evaluate the accuracy of our aging signature. P < 0.05 was considered as statistical significance.

## Supplementary Material

Supplementary Figure 1
