# Supplementary material for: The aging-related risk signature in colorectal cancer
Source: Aging (Albany NY). 2021 Feb 26;13(5):7330–49. doi: 10.18632/aging.202589 (PMC7993742; doi:10.18632/aging.202589)
Supplement: Supplementary Figure 1 [file aging-13-202589-s001.pdf]

SUPPLEMENTARY FIGURE

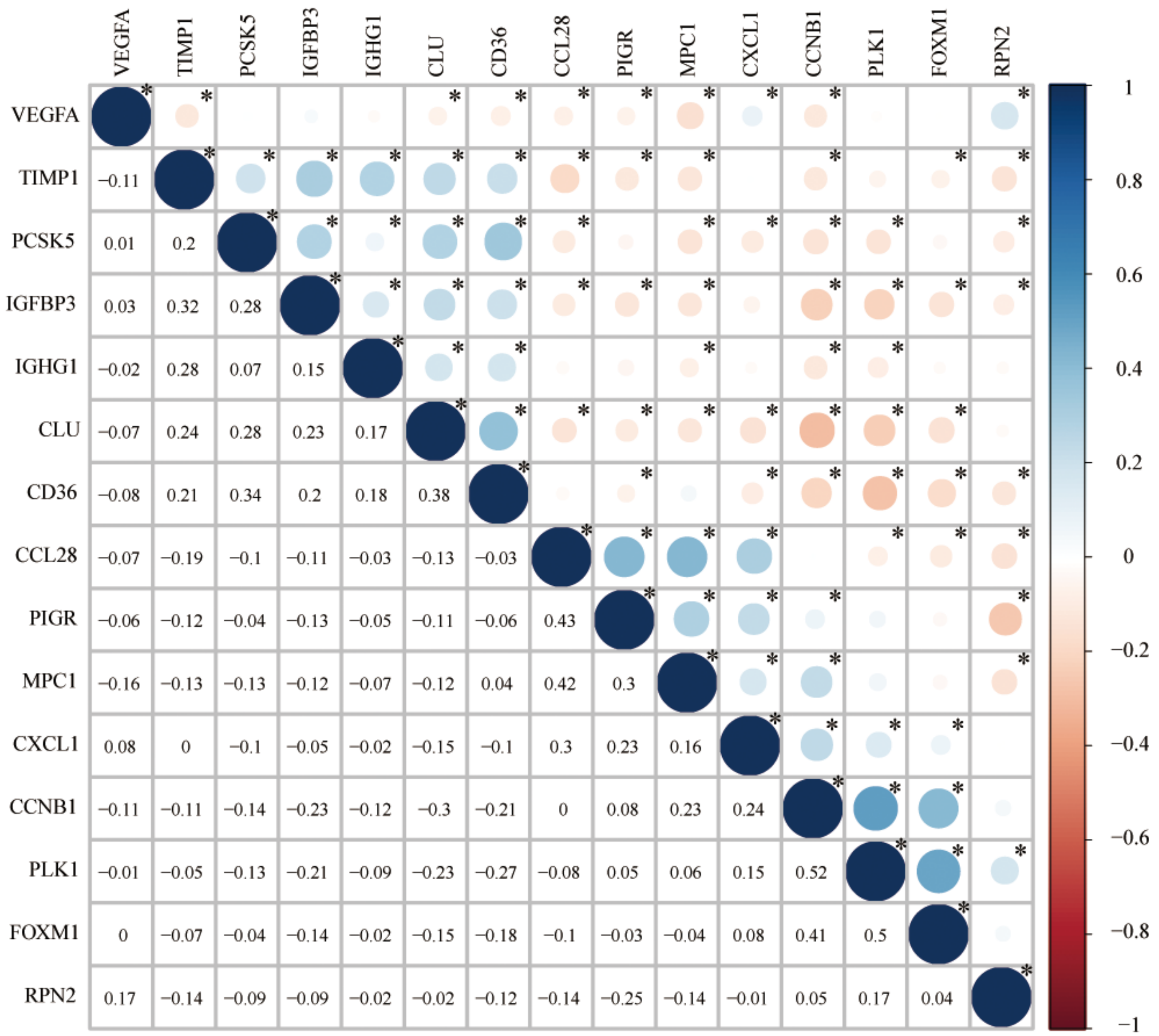

Supplementary Figure 1. Pearson correlation analysis between 15 hub genes. The bar on the left of the map indicated the legend of the Pearson correlation coefficient. Dots size and values showed the degree of their correlation, and dots color indicated whether they were positive-related (blue dots) or negative-related (red dots). * meant significance (p < 0.05).
